# A study of reproducibility of kinesiology tape applications: review, reliability and validity

**DOI:** 10.1186/s12891-019-2533-0

**Published:** 2019-04-09

**Authors:** Francisco Selva, Alberto Pardo, Xavier Aguado, Ignacio Montava, Luis Gil-Santos, Carlos Barrios

**Affiliations:** 1 Department of Physiotherapy, Own Master in evaluation, physiotherapy and reconditioning in sports science, University of Valencia, Calle Gascó Oliag 3, 46010 Valencia, Spain; 2 School of Physical Activity and Sport Sciences, University of Valencia, Calle Gascó Oliag 3, 46010 Valencia, Spain; 3 Group of Human Biomechanics and Sports, University of Castilla-La Mancha, Avenida de Carlos III s/n, 45071 Toledo, Spain; 4 Department of Textile and Paper Engineering, Valencia Polytechnic University, Plaza Ferrándiz y Carbonell, 1, 03801 Alcoy, Spain; 5 Institute for Research on Musculoskeletal Disorders, School of Medicine, Valencia Catholic University, c/ Quevedo, 2, 46001 Valencia, Spain

**Keywords:** Mechanical processes, Strain, Elongation, Adherence, Injury prevention

## Abstract

**Background:**

Literature addressing the mechanical properties of kinesiology tape is quite scarce. There are no studies which focus on the mechanical characteristics of kinesiology tape, its mechanical properties, nor its adherence following the ISO international standard test methods for tape elongation.

**Methods:**

This study quantified the mechanical characteristics of 380 samples of kinesiology tape from 19 different brands and in 4 different colors using a dynamometer. Mechanical testing was controlled by UNE EN ISO 13934-1.

**Results:**

Significant differences were found between tape brands in terms of grammage, maximum force tenacity, work, pre-elongation and percentage elongation (*P* < .001). Regarding kinesiology tape color, statistically significant differences were found between tape brands in terms of grammage, maximum force and tenacity (P < .001), work and pre-elongation (*P* < .05). When adherence was studied, statistically significant differences were found between tape brands in terms of maximum force and work (P < .001).

**Conclusions:**

The different kinesiology tapes presented different behaviors with regard to rupture and removal when applied to skin in dry state, wet state and after being submerged in artificial acidic sweat solution. Therefore, different kinesiology tape brands will produce different levels of strain even though the same elongation is used. Depending on the characteristics (body dimensions) and properties (skin elongation) of each subject in the sample, bandages with different elongations must be applied to achieve the same strain in all of the tapes and therefore produce the same effect. The absence of these data at this time limits the reliability of previous clinical studies, makes comparing their findings impossible and presents new challenges for research in this field.

## Background

Recent years have seen significant developments in bandaging techniques, above all with the appearance of kinesiology tape (KT). These tapes have a plain weave structure and, thanks to their elastane content, allow for longitudinal stretch.

Some authors credit KTs with effects such as the improvement of somatosensory stimulation and an increase in mechanoreceptive and proprioceptive impulses which cause various responses such as the facilitation or inhibition of muscle activation [1–3]. However, there is insufficient clinical evidence to support these claims [4, 5].

The application of KT has become a popular treatment among athletes, although its real effects are still being investigated [6].

Nevertheless, various authors encourage the use of KT for all athletes as a way to prevent and treat musculoskeletal injuries [7–10] or control static and dynamic posture [9, 11].

There is no clear consensus regarding the key aspects of KT application methodology, such as the percentage elongation to be used [5]. Notwithstanding Lim’s [5] results regarding percentage elongation, their review suggests that the effect size for pain reduction was lower when the studies applied more tension and left the tape in situ longer.

Consequently, KT applications cannot be reliably reproduced. According to the analysis in published systematic reviews [12–16], studies into KT present either low or very low methodological quality when assessed using the Grading of Recommendations Assessment, Development and Evaluation system (GRADE) adopted by the Cochrane Collaboration; as a result, there is currently no clinically significant evidence to support the use of KT as a therapeutic tool [17]. GRADE methodology is not a definitive fixed guide but rather provides suggestions regarding how to approach the literature, developing an optimal system of rating quality of evidence and strength of recommendations for clinical practice guidelines [18].

Even though extensive effort has been invested in evaluating the efficacy of KT, there is still a dearth of attempts to collate the findings from individual studies to determine the effects of KT application on pain and disability and, if these effects are found, their magnitude [5].

It is necessary to define standardized methodological criteria so to that effects of KT can be demonstrated. [19, 20].

Specific research studies, such as that by Pamuk and Yucesoy [21] deem the application of KT to be effective. Magnetic resonance (MR) imaging has been used to provide a reliable representation of tissue deformation, including changes in the length of muscle fibers and the direction of this change after the application of KT. The lack of homogeneity in the deformation of muscle fibers produced by KT strain indicates the occurrence of epimuscular myofascial force transmission. Accordingly, changing the level of tension the KT applies to the skin can transmit different levels of force directly to muscle tissues, either to stimulate or inhibit. Pamuk and Yucesoy [21] produced a detailed evaluation of the local tissue deformation occurring acutely under the mechanical load imposed by the application of KT. Their results show local tissue deformations produced by the effects of KT application, confirming that KT also affects non-targeted tissues and sustaining the role of a neuro-mechanical coupling in the entire limb.

Although these studies show that kinesiology tape is effective, the specific action mechanisms of KTs and their real physiological effects remain unknown [19–21].

As a result, defining the methodological characteristics of the application of kinesiology tape is deemed to be a priority.

As they do not use an agreed methodology, positive results in previous KT studies may be attributed to placebo effects, too [19].

Two studies have been published regarding the mechanical properties of KT [22, 23]. The first, by Fernández Rodríguez et al. [22], included 11 tapes from 4 brands which were analyzed and compared in terms of maximum percentage stretch, maximum force applied before rupture, thickness, density and grammage. The authors postulate that there are differences between the mechanical properties of the various brands and colors of KT but do so without completing a statistical analysis of their results.

The second study, by Matheus et al. [23], found significant differences between 50 specimens of KT (10 samples from 5 different brands or manufacturers), testing the maximum strain, maximum deformation, maximum load, and rigidity using an EMIC universal testing machine (model DL 10.000). They also found significant differences in adherence force when removing KT specimens from a metal plate.

There are no studies into the characteristics of KT, its mechanical properties, maximum adherence force and work done when removing it from skin in dry, wet, or sweaty state (the state of the skin will affect the tape’s adherence) which follow ISO international standard test methods for bandage stretching [24]. Following standard test methodology is necessary to enable KT applications to be reproduced.

The reproducibility of the effect of KTs is crucial in clinical settings. It is possible that the effect the application produces on the tissues may differ depending on the mechanical properties of each tape. Perhaps the absence of any effect from the application of KT reported in previous studies is related to the characteristics of the different KTs and the application time.

Consequently, to repeatedly obtain a certain level of strain there would have to be no variation in the properties of the tape. The reproducibility of the effects of KT has not been studied with sufficient rigor until now.

Our objective was to determine if KTs have different characteristics and mechanical properties in terms of rupture and adherence in dry state, wet state and after being submerged in artificial acid sweat solution. The tapes studied were grouped by color and brand to standardize the KT applications. This analysis will facilitate the reproducibility and standardization of KT application strain through knowledge of the different elongation percentages of each KT, thereby facilitating methodologically correct tape applications to achieve reproducible effects and so determine the limitations of KT.

## Methods

### Specimens

The characteristics and mechanical rupture properties, as well as the adherence properties of 380 specimens from 19 brands of KT were analyzed. Rolls of tape in four different colors (reference 1: blue; reference 2: black; reference 3: beige; reference 4: red) from each brand were tested. All the KTs were new, unused and unopened.

### Study design

**Fig. 1:**
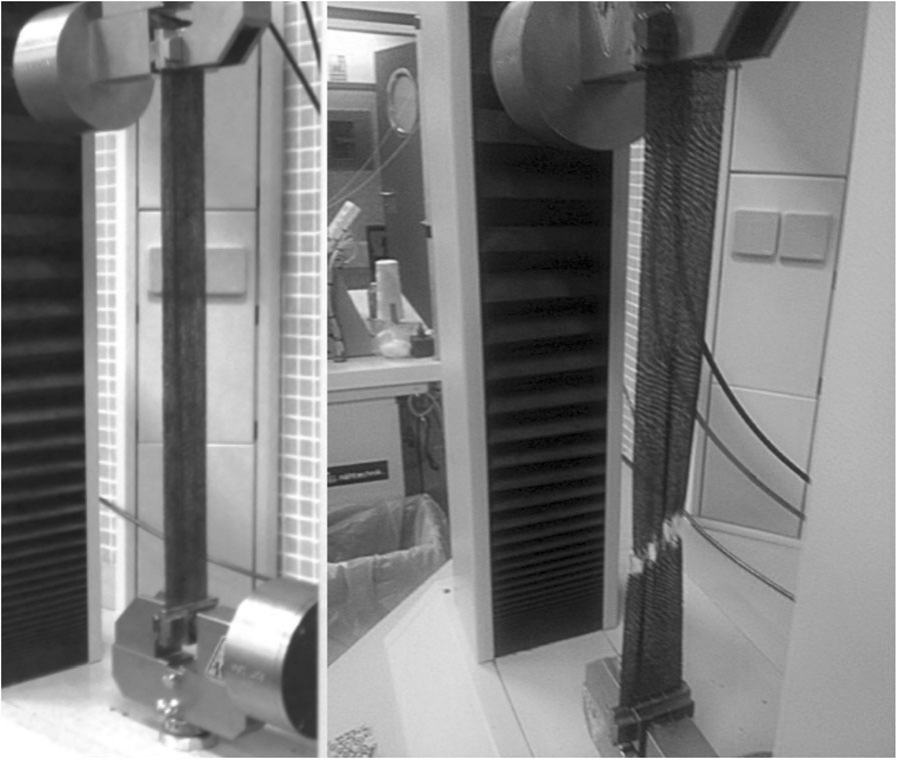
Dynamometer Zwick/Roell. model Z005 (Ulm. Germany) during the strain test of one black tape. The appearance of white marks in the tape indicates its immediate breakage

A Z005 dynamometer (Zwick Roell, Ulm, Germany) (Fig. 1), controlled by the V10.11 ZwickTestXpert software, was used for the mechanical testing. Before beginning each test, the dynamometer software requested the grammage of each specimen to adjust the preload. A 5 cm^2^ sample of each KT was used for this purpose. The ISO standard practice for atmosphere conditioning and physical testing of textiles [24] was followed, as was the ISO standard practice for preconditioning and conditioning the tests [25].

ISO standard testing practice [26] specifies a reliable procedure to determine the maximum force and elongation at maximum force of textiles using the strip method.

Following ISO standard testing practice [24], the specimens were 300 mm in length and 50 mm in width. They were stretched at a constant rate of extension until rupture occurred. The mean value of the data obtained was calculated.

The characteristics tested were: the elongation under which the tape adhered to the backing paper or pre-elongation capacity, and grammage (weight in g / cm^2^).

The mechanical properties tested were: maximum force (N or Kg); tenacity (kg / mm^2^); work (kg / mm); elongation without paper (%).

To evaluate the adherence force and the work done to remove the KT from skin, pieces of untanned sheepskin, 50-mm wide by 80-mm long, were included in the test. 50 mm of one end of the KT sample were stuck to the piece of skin at 0% elongation leaving 30 mm of skin at one end of the piece uncovered.

**Fig. 2:**
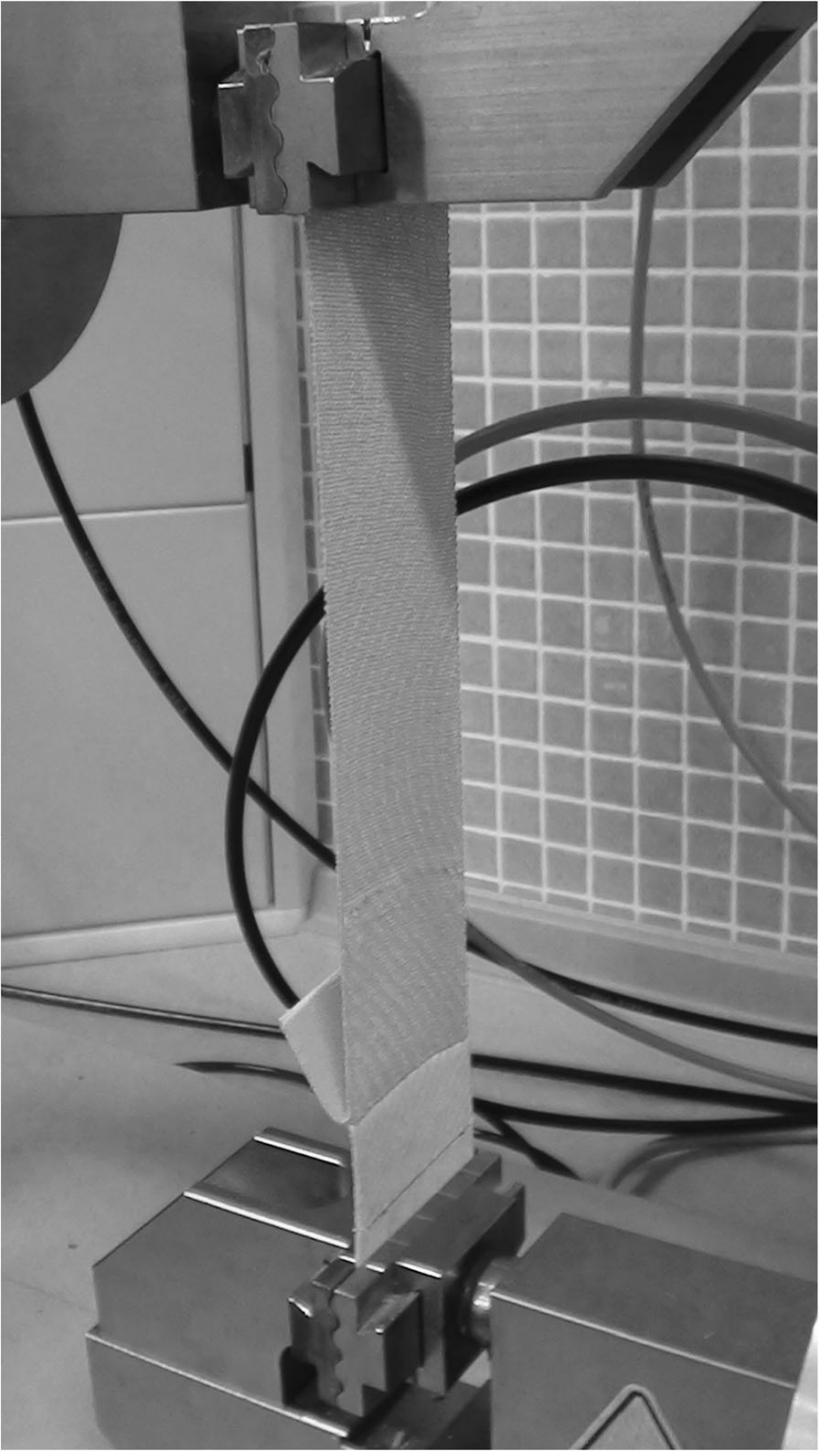
Dynamometer Zwick/Roell. model Z005 (Ulm. Germany) during the adherence test of one red tape. The tape is adhered on a piece of untanned sheepskin

Subsequently, to carry out the adherence test in dry state, 20 mm of the uncovered skin was secured in the lower dynamometer clamp, leaving 10 mm of uncovered skin between the clamp and the KT. The backing paper was removed from the KT and, at 0% elongation, the end of the tape not adhered to the skin was secured in the upper clamp while leaving 40 mm of tape between the skin piece and the upper clamp. The distance between the clamps was 100 mm (Fig. 2).

The KT was preloaded by stretching it without removing it from the skin until ±2 N of strain were reached. From this moment, the dynamometer began to separate the clamps, stretching the test piece until it came away from the skin.

To carry out the tests in wet state, water quality parameters for Grade 3 water were used, in accordance with ISO specifications for water for analytical laboratory use [27]. The water was poured into a suitable, clean, airtight vessel. Having previously stabilized the adherence force, the skin samples with attached KT were submerged in the solution for 10 min. After this time had lapsed the previously described dynamometer methodology for testing the dry samples was repeated with the wet samples.

To carry out the tests with artificial acidic sweat, the formula for artificial acidic sweat defined in the relevant UNE standard [28] was used. Having previously stabilized the adherence force, the samples were submerged for 10 min in said solution. After this time, the previously described dynamometer methodology was repeated for these samples.

Components extracted from 7 black-color specimens were also analyzed by gas chromatography–mass spectrometry (GC-MS). An Agilent 5973 N mass spectrometer with a low resolution quadrupole analyzer was used, with an Agilent 6890 N gas chromatograph for capillary columns (split/splitless, pulsed split and pulsed splitless) and GC-MS interface. An Agilent 7683 Automatic Liquid Sampler and a large NIST spectroscopy database were also used. Four equally-sized pieces were cut from each of the different KTs and placed in 15 ml of dichloromethane. The solution was agitated for 1 h at room temperature. 1 ml of the solution was filtered and injected into the chromatograph.

### Statistical analysis

The data were analyzed using the SPSS Statistics 20 program (IBM, Spanish version). The distribution of the data was evaluated using the Kolmogorov-Smirnov test. We also prepared descriptive statistics for each variable (mean ± standard deviation). One-way (between the subjects) ANOVA was used for the mean differences test. The significant differences were investigated further using pairwise comparisons to control inflation of type I errors, specifically the Tukey tests. Before the analyses were carried out, the parametric assumptions such as approximate normality and homogeneity of variances, and these detection analyses, were considered not to present any impediment to the use of ANOVA. The alpha value = 0.05 was used as the significance level and the confidence interval for the mean value was 95%.

## Results

### Rupture

#### Grouped by brand

The mean values, standard deviations, and differences in the grammage of the specimens are presented in Table 1, grouped by brand. Brand was found to have a statistically significant effect based on grammage (F = 13.56; *P* < .001). The grammage ranged from 179.1 to 230.5 g / m^2^.

**Table 1:** Companies/Brand, patent, country of origin, grammage and results of the different mechanical properties at break of the tapes for brands. Data are expressed as mean (standard deviation)

Reference	Companies/Brand Name	Patent	Country	n	Grammage (g/m^2^)	Differences (*)	Peak force (kg)	Differences (*)	Tenacity (kg/mm)	Differences (*)	Work (kg/mm)	Differences (*)	Elongation (%)	Differences (*)	Pre- elongation (%)	Differences (*)
1	Balance Tape™	Balance Taping Europe AB	Sweden	20	192.81 (13.54)	2, 5, 7, 8, 12, 15, 16, 18, 19	18.23 (1.20)	3, 8, 10	0.37 (0.02)	3, 8, 10	0.38 (0.07)	4, 12–16, 18	49.49 (9.03)	2, 4, 6, 12, 13, 15–18	7.00 (0.70)	3, 18
2	K Tape©	K-Taping	Germany	20	212.35 (5.06)	1, 5, 6, 10	17.08 (1.62)	3, 8, 18	0.34 (0.03)	3, 8, 18	0.46 (0.06)	3, 6, 10	65.30 (1.75)	1, 6, 7, 8, 9, 10, 11	5.33 (0.41)	3, 12
3	Leukotape® K	BSN medical	Germany	20	202.20 (20.36)	5, 6, 7, 16, 18	12.30 (1.11)	4, 6, 8, 9, 11 19	0.25 (0.02)	1, 2, 4, 6, 8, 9, 11–19	0.30 (0.04)	2, 4, 8, 12–19	56.34 (993)	6, 10, 12	10.86 (1.76)	1, 2, 4, 5, 6, 7–19
4	Kinsiotape™	Visiocare	Italy	20	206.86 (28.35)	5, 6, 10	18.80 (3.24)	8, 10	0.37 (0.06)	3, 8, 10	0.51 (0.18)	1, 3, 5, 6, 7, 10	62.89 (17.58)	1, 6, 7, 8, 10	6.50 (1.01)	3, 18
5	KT Tape®	Lumos. Inc	USA	20	230.45 (4.88)	1–4, 6, 9, 10, 11, 13, 14, 17	16.22 (0.97)	8, 13, 16, 18,19	0.32 (0.01)	8, 13, 16, 18, 19	0.38 (0.03)	4, 12–16, 18	55.40 (3.68)	6, 10, 12, 16	6.76 (0.32)	3, 18
6	K-active®	K-Active Europe GmbH	Germany	20	179.05 (14.36)	2–5, 8, 9, 11–19	18.40 (1.72)	8, 10	0.37 (0.03)	3, 8, 10	0.31 (0.04)	2, 4, 8, 12–19	36.90 (2.34)	1, 2, 3, 4, 5, 7–19	7.94 (0.17)	8, 18
7	Kinesiologic Tape-Original™	Tape-Original	Germany	20	222.30 (9.36)	1, 3, 6, 10, 17	16.15 (1.23)	8, 13, 16, 18, 19	0.32 (0.02)	8, 13, 16, 18, 19	0.35 (0.03)	4, 8, 12–16, 18, 19	49.01 (3.79)	2, 4, 6, 12, 13, 15–18	6.44 (0.30)	3, 18
8	Energy-Ion™ Kinesiology Tape	OC Group	Spain	20	215.37 (13.54)	1, 6, 10	26.03 (7.33)	All brands	0.52 (0.14)	All brands	0.49 (0.14)	3, 6, 7, 10	49.22 (5.47)	2, 4, 6, 12, 13, 15–18	4.44 (0.28)	3, 6, 12, 14, 16
9	Kinesio® Tex Golg™	Kinesio Taping	USA	20	207.79 (17.76)	5, 6, 10	17.78 (2.12)	18	0.36 (0.04)	3, 8, 18	0.40 (0.08)	10, 12, 13, 15, 16, 18	54.70 (9.41)	2, 6, 10, 12, 15, 16	5.47 (0.32)	3
10	Kinesiology Tape™	Temtex	South Korea	20	186.44 (5.17)	2, 4, 5, 7, 8, 9, 11–19	14.17 (2.00)	11–19	0.28 (0.03)	1, 4, 6, 8, 11–19	0.27 (0.03)	2, 4, 8, 9, 11–19	41.81 (1.84)	2–5, 9, 11–19	6.80 (0.42)	3, 18
11	Rock Tape®	Rock Tape	USA	20	206.62 (10.75)	5, 6, 10, 16	18.22 (2.30)	3, 8, 10	0.37 (0.04)	3, 8, 10	0.40 (0.08)	10, 12, 13, 15, 16, 18	53.74 (10.61)	2, 6, 10, 12, 15, 16	4.97 (0.21)	3, 6, 12, 14, 16
12	Cure Tape™	Fysiotape	Netherlands	20	214.00 (4.59)	1, 6, 10	19.34 (1.37)	3, 8, 10	0.39 (0.02)	3, 8, 10	0.53 (0.04)	1, 3, 5, 6, 7, 9–11	68.49 (2.67)	1, 3, 5–11, 14, 19	8.04 (0.43)	2, 3, 8, 11, 18
13	Mueller® Tape	Mueller Sports Medicine. Inc.	USA	20	207.84 (10.77)	5, 6, 10	20.85 (1.74)	3, 5, 7, 8, 10	0.42 (0.03)	3, 5, 7, 8, 10	0.55 (0.05)	1, 3, 5, 6, 7, 9–11	62.90 (6.12)	1, 6, 7, 8, 10	6.94 (0.58)	3, 18
14	Sports Tex™	Atextaping	South Korea	20	208.76 (18.93)	5, 6, 10	19.01 (2.02)	3, 8, 10	0.38 (0.04)	3, 8, 10	0.50 (0.10)	1, 3, 5, 6, 7, 10	57.01 (11.07)	6, 10, 12	7.74 (1.65)	3, 8, 11, 18
15	Energy-Ion™ Kinesiology tape with Turmalina	OC Group	Spain	20	215.12 (12.92)	1, 6, 10	19.99 (1.58)	3, 8, 10	0.40 (0.03)	3, 8, 10	0.54 (0.04)	1, 3, 5, 6, 7, 9–11	65.12 (4.52)	1, 6–11	6.20 (0.33)	3, 18
16	BB Tape™	Altermed Co. Lld.	South Korea	20	223.76 (5.67)	1, 3, 6, 10, 11, 17	20.50 (1.88)	3, 5, 7, 8, 10	0.41 (0.03)	3, 5, 7, 8, 10	0.56 (0.07)	1, 3, 5–11	66.09 (2.56)	1, 5–11	7.60 (0.17)	3, 8, 11, 18
17	Kinesiology Tape Irisana™ Turmalina	Irisana S.A	Spain	20	205.00 (17.87)	5, 6, 7, 10, 16	18.39 (1.69)	3, 8, 10	0.37 (0.03)	3, 8, 10	0.44 (0.11)	3, 6, 10	60.45 (17.32)	1, 6, 7, 8, 10	6.60 (0.60)	3, 18
18	Kinesiology Tape Sarasa™	Sarasa	Japan	20	219.70 (32.13)	1, 3, 6, 10	21.85 (10.92)	2, 3, 5, 7, 8, 9, 10	0.44 (0.21)	2, 3, 5, 7, 8, 9,10	0.5 (0.28)	1, 3, 5, 6, 7, 9–11	63.21 (17.08)	1, 6, 7, 8, 10	3.00 (0.25)	1, 3–7, 10, 12–17
19	No brand name	No patent	South Korea	20	208.99 (19.32)	1, 6, 10	20.93 (2.57)	3, 5, 7, 8, 10	0.42 (0.05)	3, 5, 7, 8, 10	0.49 (0.08)	3, 6, 7, 10	56.23 (4.99)	6, 10, 12	5.56 (0.12)	3

(*) This column indicates the significant differences between that brand and the brands corresponding to the different numbers (at least P < .05). Each brand was statistically different in properties to the other brands

Statistically significant differences were found in all tape parameters grouped by brand: maximum force (F = 14.20; *P* < .001), tenacity (F = 14.10; *P* < .001), work (F = 15.01; *P* < .001), pre-elongation (F = 10.05; *P* < .001), and percentage elongation (F = 17.46; *P* < .001). The data are presented in Table 1.

Maximum force ranged from 12.3 to 26.0 kg, tenacity from 0.246 to 0.520 kg / mm, work from 0.27 to 0.56 kg / mm, pre-elongation capacity from 4.4 to 10.9% and percentage elongation from 36.9 to 68.5%.

Brand 8 demonstrated the greatest maximum force and tenacity, demonstrating a statistically significant difference from the other brands.

#### Grouped by KT color

There were significant differences in grammage (F = 6.50; *P* < .001) between colors of KT.

Statistically significant differences were found between the different colored tapes with regard to maximum force (F = 15.07; *P* < .001), tenacity (F = 15.22; *P* < .001), work (F = 7.04; *P* < .05), and pre-elongation (F = 12.97; *P* < .05). There was no significant difference in percentage elongation (F = 1.14; *P* > .05).

Black KT demonstrated the lowest maximum force capacity, tenacity and pre-elongation. Blue KT demonstrated the greatest work capacity and the greatest pre-elongation. The differences between the other colored tapes were not statistically significant. Black KTs had the highest grammage.

### Chemical components of the adhesive

The results showed that the primary substances contained in and common to all the KT tested were: Cyclotrisiloxane, hexamethyl; Cyclotrisiloxane, octamethyl; Limonene/cyclohexanol and 1-hexanol. Subsequently, 58 substances were recovered which were contained in at least one of the KTs studied.

### Adherence in dry state

#### Grouped by brand

Statistically significant differences were found between tape brands in terms of maximum force (F = 55.85; *P* < .001) and work (F = 10.71; *P* < .001).

Maximum force ranged from 0.85 to 4.31 kg (95% CI: 2.11–2.29) and work from 0.11 to 0.60 kg / mm (95% CI: 0.14–0.17) (Table 2).

**Table 2:** Reference grouped by brand. Adherence in dry state. Adherence in wet state and Adherence with artificial perspiration solution. Peak force and work testing. Data are expressed as mean (standard deviation)

	Adherence in dry state								Adherence in wet state								Adherence with artificial perspiration solution							
	Peak force (kg)				Work (kg/mm)				Peak force (kg)				Work (kg/mm)				Peak force (kg)				Work (kg/mm)			
Reference	Mean	SD	Least	Greatest	Mean	SD	Least	Greatest	Mean	SD	Least	Greatest	Mean	SD	Least	Greatest	Mean	SD	Least	Greatest	Mean	SD	Least	Greatest
1	2.14	0.49	1.46	3.08	0.14	0.07	0.02	0.24	1.38	0.34	0.89	2.10	0.07	0.03	0.02	0.15	0.65	0.27	0.46	1.42	0.03	0.01	0.01	0.05
2	1.77	0.24	1.32	2.12	0.01	0.01	0.01	0.01	1.29	0.34	0.93	2.12	0.01	0.01	0.01	0.01	0.69	0.20	0.51	1.06	0.01	0.01	0.01	0.01
3	2.89	0.24	2.37	3.24	0.16	0.12	0.04	0.36	1.33	0.24	0.95	1.67	0.06	0.04	0.01	0.16	0.61	0.10	0.47	0.79	0.03	0.01	0.01	0.06
4	2.32	0.20	1.91	2.69	0.19	0.08	0.08	0.32	1.42	0.36	0.66	2.13	0.05	0.02	0.01	0.11	1.00	0.41	0.34	1.67	0.01	0.01	0.01	0.01
5	3.53	0.55	2.61	4.31	0.19	0.11	0.04	0.41	1.20	0.46	0.73	1.98	0.04	0.03	0.01	0.10	0.96	0.50	0.22	1.84	0.01	0.01	0.01	0.02
6	1.62	0.23	1.22	2.07	0.01	0.01	0.01	0.01	0.67	0.11	0.49	0.84	0.01	0.02	0.01	0.07	0.36	0.04	0.29	0.47	0.01	0.01	0.01	0.03
7	1.73	0.12	1.53	1.95	0.13	0.06	0.02	0.19	1.00	0.08	0.82	1.10	0.01	0.01	0.01	0.01	0.52	0.10	0.39	0.71	0.01	0.01	0.01	0.01
8	2.01	0.26	1.42	2.46	0.14	0.05	0.05	0.26	1.01	0.23	0.64	1.26	0.02	0.01	0.01	0.05	0.66	0.20	0.41	1.07	0.01	0.01	0.01	0.01
9	2.97	0.25	2.30	3.21	0.22	0.08	0.04	0.36	0.62	0.18	0.45	1.12	0.04	0.03	0.01	0.11	0.47	0.11	0.31	0.67	0.01	0.01	0.01	0.04
10	2.69	0.32	2.24	3.26	0.12	0.03	0.04	0.16	0.65	0.18	0.35	0.90	0.03	0.02	0.01	0.06	0.54	0.11	0.39	0.81	0.01	0.01	0.01	0.01
11	2.34	0.28	1.95	2.84	0.16	0.06	0.05	0.28	0.99	0.21	0.70	1.41	0.06	0.03	0.01	0.13	0.63	0.15	0.43	0.86	0.01	0.01	0.01	0.02
12	2.36	0.25	2.00	2.81	0.22	0.04	0.14	0.26	0.66	0.19	0.41	1.07	0.04	0.01	0.01	0.09	0.45	0.17	0.28	0.90	0.03	0.02	0.01	0.07
13	2.34	0.27	1.84	2.88	0.16	0.03	0.14	0.24	0.64	0.15	0.34	0.87	0.04	0.01	0.01	0.07	0.28	0.05	0.21	0.36	0.01	0.01	0.01	0.03
14	2.85	0.30	2.36	3.19	0.18	0.03	0.15	0.25	0.99	0.20	0.65	1.32	0.03	0.02	0.01	0.08	0.51	0.08	0.38	0.65	0.01	0.01	0.01	0.03
15	2.56	0.40	1.87	3.08	0.25	0.11	0.16	0.60	0.95	0.10	0.79	1.12	0.02	0.01	0.01	0.06	0.49	0.24	0.20	0.85	0.01	0.01	0.01	0.02
16	1.58	0.04	1.51	1.68	0.23	0.07	0.17	0.45	0.68	0.07	0.54	0.82	0.01	0.01	0.01	0.02	0.39	0.04	0.34	0.48	0.01	0.01	0.01	0.02
17	1.41	0.09	1.21	1.51	0.13	0.01	0.11	0.17	0.45	0.05	0.36	0.52	0.01	0.01	0.01	0.01	0.21	0.06	0.14	0.32	0.01	0.01	0.01	0.01
18	1.36	0.11	1.23	1.54	0.12	0.01	0.11	0.14	0.40	0.09	0.19	0.53	0.01	0.01	0.01	0.01	0.17	0.04	0.12	0.24	0.01	0.01	0.01	0.01
19	1.34	0.24	0.85	1.70	0.10	0.05	0.01	0.18	0.45	0.11	0.30	0.67	0.01	0.01	0.01	0.03	0.20	0.05	0.13	0.29	0.01	0.01	0.01	0.01

*SD* Standard deviation

#### Grouped by KT color

A one-way ANOVA test showed no statistically significant differences between the KTs grouped by color in terms of maximum force (F = 0.41; *P* > .05) or work (F = 0.91; *P* > .05). Blue KT demonstrated the greatest maximum force and Tape 4 the least. Black KT demonstrated the greatest work and Red KT the least (Table 3).

**Table 3:** Reference grouped by KT color. Adherence in dry state. Adherence in wet state and Adherence with artificial perspiration solution. Peak force and work testing. Data are expressed as mean (standard deviation)

	Adherence in dry state								Adherence in wet state								Adherence with artificial perspiration solution							
	Peak force (kg)				Work (kg/mm)				Peak force (kg)				Work (kg/mm)				Peak force (kg)				Work (kg/mm)			
Color	Mean	SD	Least	Greatest	Mean	SD	Least	Greatest	Mean	SD	Least	Greatest	Mean	SD	Least	Greatest	Mean	SD	Least	Greatest	Mean	SD	Least	Greatest
Blue	2.23	0.71	1.15	4.31	0.16	0.09	0.01	0.45	0.86	0.33	0.36	2.10	0.03	0.02	0.01	0.13	0.49	0.21	0.12	1.17	0.01	0.01	0.01	0.05
Black	2.23	0.66	1.03	4.26	0.16	0.10	0.01	0.60	0.91	0.40	0.34	1.98	0.03	0.02	0.01	0.15	0.46	0.25	0.13	1.34	0.01	0.01	0.01	0.06
Beige	2.23	0.66	1.21	4.13	0.15	0.08	0.01	0.31	0.91	0.45	0.19	2.12	0.03	0.03	0.01	0.11	0.56	0.37	0.13	1.84	0.01	0.01	0.01	0.05
Red	2.12	0.62	0.85	3.43	0.14	0.08	0.01	0.28	0.83	0.36	0.30	2.13	0.02	0.03	0.01	0.16	0.55	0.30	0.12	1.67	0.01	0.01	0.01	0.07

*SD* Standard deviation

### Adherence in wet state

#### Grouped by brand

Statistically significant differences were found between the KT brands in terms of maximum force (F = 25.61; *P* < .001) and work (F = 7.71; *P* < .001).

Maximum force ranged from 0.19 to 2.13 kg (95% CI: 0.83–0.93) and work from 0.025 to 0.168 kg / mm (95% CI: 0.029–0.037) (Table 2).

#### Grouped by KT color

A one-way ANOVA test showed no statistically significant differences between the KTs grouped by color in terms of maximum force (F = 0.56; *P* > .05) or work (F = 0.71; P > .05). Red KT demonstrated the greatest maximum force and Beige KT the least. Red KT demonstrated the greatest work and Beige KT the least (Table 3).

### Adherence with artificial perspiration solution

#### Grouped by brand

Statistically significant differences were found between the KT brands in terms of maximum force (F = 15.39; *P* < .001) and work (F = 12.04; *P* < .001).

Maximum force ranged from 0.12 to 1.84 kg (95% CI: 0.48–0.55) and work from 0.001 to 0.718 kg (95% CI: 0.001–0.015) (Table 2).

#### Grouped by KT color

A one-way ANOVA test showed no statistically significant differences between the KTs grouped by color in terms of maximum force (F = 1.51; *P* > .05) or work (F = 1.08; P > .05). Beige KT demonstrated the greatest maximum force and Blue KT and Red KT the least. Red KT demonstrated the greatest work and Beige KT the least (Table 3).

## Discussion

The objective of this study was to define the characteristics, and mechanical rupture and adherence properties of a wide variety of KT specimens. In this way, we attempt to respond to the need to define standardized and reproducible application criteria so that the effects of KTs can be specified.

In the previous studies we analyzed, the values described for the maximum elongation, expressed as a percentage of the specimen’s initial elongation, ranged from 20 to 40% [20, 29–31] or 55–60% [32] to 120–140% [1, 33–37], 140–150% [38] and 250 to 400% [23]. None of the 380 specimens tested reached an elongation of 100% of their initial length (600 mm) before rupture.

The two previous studies [22, 23] found differences between their specimens, and [23] found significant differences between the KT brands tested. This study revealed considerable variation between the different brands and colors of KT in terms of maximum force, tenacity, work, pre-elongation and percentage elongation y grammage (Table 1). Following the standard testing methodology, the grammage of each specimen must be taken and a pre-load must be carried out before each test. The previous study which used a dynamometer [23] does not mention this information and consequently the method used to extract the data remains unknown. This makes it impossible to reproduce the research and could lead to the generation of further conflicting results.

Untanned sheepskin was used to ensure that the adherence test was as reliable as possible and its results were applicable to clinical practice. The maximum adherence force and work done when removing the tape from the skin in dry state was very high, lower when the skin had been submerged in an aqueous solution, and even lower when it had been submerged in an artificial acidic sweat solution (Tables 2 and 3). Study [23] obtained far lower values using a metal plate.

**Fig. 3:**
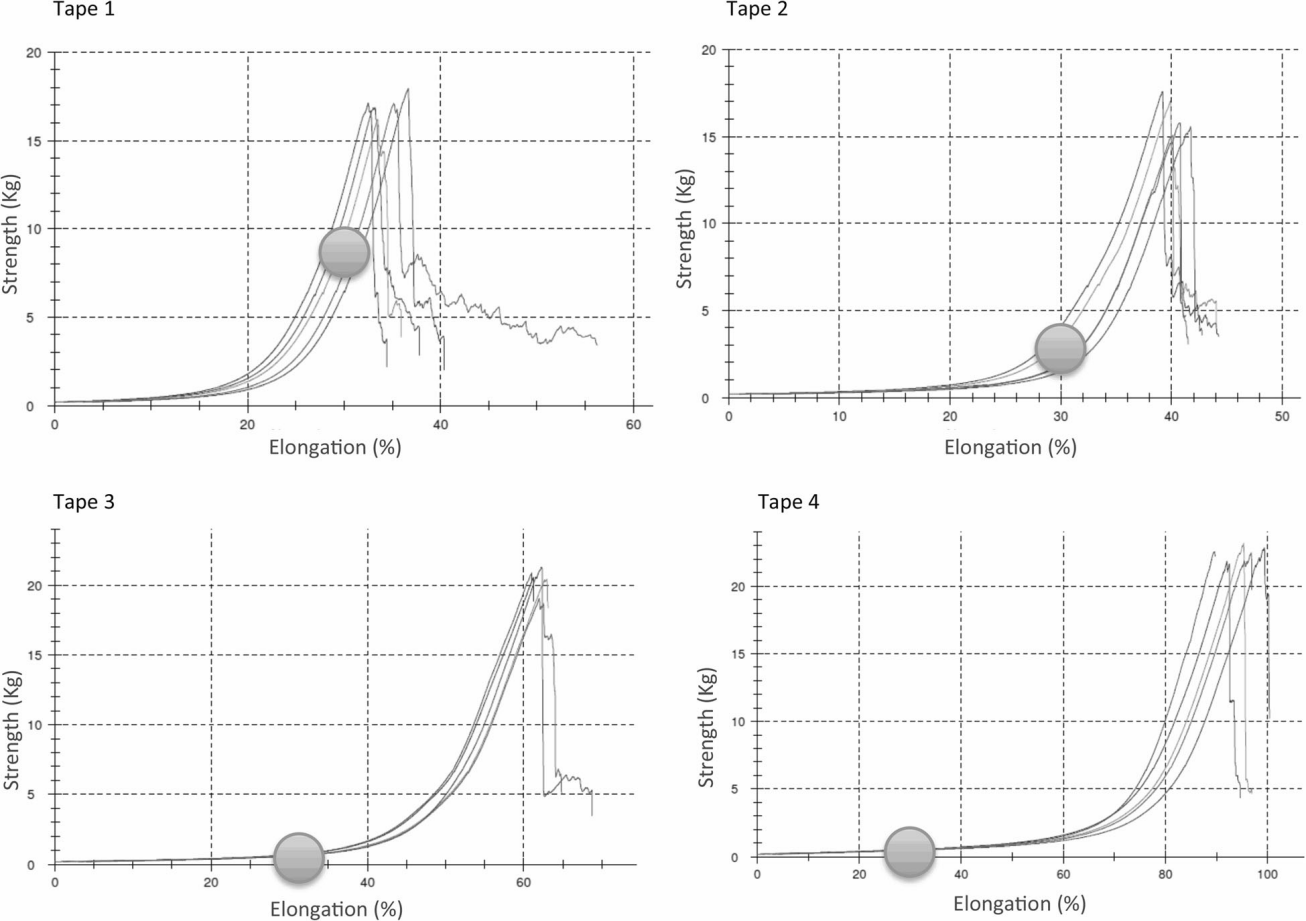
Tape 1: Strain of a beige tape (reference [10]) until breakage. Maximum elongation is 33–37%. requiring 16 to 18 kg of strength to breakage. Tape 2: Strain of a red tape (reference [9]) until breakage. Maximum elongation is 37–45%. requiring 16 to 17 kg of strength to breakage. Tape 3: Strain of a blue tape (reference [11]) until breakage. Maximum elongation is 60–63%. requiring 21 to 22 kg of strength to breakage. Tape 4: Strain of a black tape (reference [4]) until breakage. Maximum elongation is 94–99%. requiring 23 to 24 kg of strength to breakage. The blue point marks the 30% elongation of each tape

Taking the maximum elongation of 4 KT specimens (Fig. 3) as a reference, they can be seen to be at different deformation points. Tape 4 will generate lower levels of strain because 30% elongation is within the material’s elastic deformation range and its flexibility means it will stretch considerably without limiting body movements. At its maximum elongation (point of rupture) the tape has lower energy absorption potential as it is in the plastic-elastic region of the stress-strain curve, tenacity is lower and, consequently, the work or total area under the curve is very large (Fig. 3). This reasoning can be considered fundamental and demonstrates that the tension produced by different tapes does vary.

We concur with Pamuk and Yucesoy [21] that the application of KT produces effects and that different applications cause different effects, but in order to specify said effects the same mechanical response must be achieved using an optimum, specific and reproducible level of tension. Achieving said mechanical response may require different elongations in different KTs. To this end the characteristics and properties of the KT used must be specified in addition to its maximum adherence force. It is also necessary to know the elongation reference used, the work done when removing the tape from the skin, the skin conditions, and the elongation of the skin to be bandaged to produce the same tension in each patient to produce the same effect (*P* < .05) without exceeding the elastic elongation of the KT both at the point of application and once the movement to be trialed has been completed.

This would facilitate the objective discovery of the different action mechanisms of KTs which could affect clinical results, as González-Iglesias et al. [39] believe.

In their literature review Morris et al. [14] only included KT studies which used the Kinesio® Tex brand. This exclusivity was to improve the accuracy of their conclusions, as they believed other brands of KT used in clinical practice were different.

It remains to be shown whether the variability in the tension and adherence of KTs changes the effect they produce during their application even when the same application metholodolgy is used for different tapes.

Our results suggest that the basic concepts of KT application are not yet complete and that studies which tried to determine the effects of KT are not reproducible. This may explain the disparity in the results obtained by different studies investigating the effects of KT. According to Lim’s review [5], there are studies that did not report the amount of stress applied while others did not report the length of each tape used in situ.

This study is limited by testing using a uniform strain and a constant velocity. Moreover, there may be a certain degree of variability between the KTs and the different brands due to them coming from different production lots.

## Conclusions

The KT specimens studied had the same structure but different chemical compositions, characteristics and mechanical properties.

The different KTs presented different behaviors with regard to rupture and removal when applied to skin in dry state, wet state and after being submerged in artificial acidic sweat solution.

If the elongation limits of each KT are unknown, many of them will produce different levels of strain even though the same elongation is used.

The absence of these data limits the reproducibility and the reliability of clinical studies using KT. Clinical studies should specify the reference used (tape length or tape elongation) to define the percentage elongation employed, something which has not been specified in studies to date.

In order for future clinical studies to be standardized and reproducible, the physiotherapist must achieve the same effect with each KT application, taking into account the reaction of its mechanical rupture and adherence properties, as well as the stretching of the skin to be bandaged.

Depending on the characteristics (body dimensions) and properties (skin elongation) of each subject in the sample, bandages with different elongations must be applied to achieve the same strain in all of them and therefore produce the same effect.

Adapting the analysis offered by MRI, the dynamometer and other devices for the study of KT would facilitate the definition of protocols to carry out highly-important studies to promote understanding of the specific mechanisms of action of KTs, their real physiological effects and the possible clinical and sporting outcomes of their use.
